# Editorial: Advances in genetics and molecular diagnosis in colorectal, stomach, and pancreatic cancer

**DOI:** 10.3389/fonc.2022.1104671

**Published:** 2022-12-19

**Authors:** Carlos Alberto Vaccaro, Julieta Soarez, Mev Dominguez-Valentin, Walter Hernán Pavicic

**Affiliations:** ^1^ Instituto de Medicina Traslacional e Ingeniería Biomédica (IMTIB) Hospital Italiano de Buenos Aires, Instituto Universitario Hospital Italiano de Buenos Aires, Consejo Nacional de Investigaciones Científicas y Técnicas (HIBA-IUHI-CONICET), Ciudad Autónoma de Buenos Aires (CABA), Argentina; ^2^ Programa de Cáncer Hereditario (Pro.Can.He.), Hospital Italiano de Buenos Aires (HIBA), Ciudad Autónoma de Buenos Aires (CABA), Argentina; ^3^ Department of Tumor Biology, Institute of Cancer Research, The Norwegian Radium Hospital, Oslo, Norway

**Keywords:** gastrointestinal cancer, colorectal cancer, pancreatic cancer, genetic predisposition, hereditary syndrome

Gastrointestinal (GI) cancer accounts for over one-quarter (26%) of all cancer cases and over one-third (35%) of cancer deaths globally (1). According to worldwide incidence and mortality rates, the most common cancers include colorectal, stomach, liver, esophageal, and pancreatic cancers (1). Although their incidence has decreased, this group of malignancies continues to pose major challenges to public health. Driven by population growth and aging worldwide, it is estimated that the number of new cases and deaths will increase by 58% and 73%, respectively, by 2040 (1).

Cancer is a complex, heterogeneous, and multifactorial disease, in which both genetic and non-genetic factors are involved. A complete identification of the risk factors, and an understanding of their role at different stages of tumor development, is essential for achieving high-quality comprehensive cancer care. In recent years, the overwhelming revolution in omics technologies, from genomics to transcriptomics, has expanded our understanding of the molecular pathogenesis of GI cancers (2). Despite the advances made in this field, extensive work is still needed to fully understand the molecular and genetic nature that leads to the development of such cancers.

This Research Topic focuses attention on advances in genetic predisposition and novel biomarkers linked to GI cancers, seeking to bring some light to this research field.

Colorectal cancer (CRC) is the most frequently diagnosed GI malignancy, and it is estimated that up to ~35% of all cases are associated with an inherited form attributable to moderate and high penetrance germline genetic alterations (2, 3). Lynch syndrome and familial adenomatous polyposis (FAP) syndrome are the two most common disorders, accounting for 2-4% and 1% of all CRCs, respectively (4). Although new predisposition genes for FAP have been identified recently, to date, up to 20% of patients with a clear FAP phenotype and as much as 80% with the attenuated form remain genetically unexplained (Olkinuora et al.). Olkinuora et al. confirmed the importance of extending the coverage of genomic studies by NGS to different populations to achieve the goal of identifying new genes that predispose to hereditary CRC. A recurrent variant in the DNA glycosylase gene *NEIL1* was observed in two non-related families from Finland and Argentina, suggesting that pathogenic germline variants in this gene can explain polyposis in FAP cases without *APC* pathogenic alterations. To date, for certain genes, such as *BRCA1*, the existing data to prove their role in CRC development are contradictory. In that regard, the article by Freire et al. reported three non-related CRC cases (families) carrying a heterozygous pathogenic germline variant in the *BRCA1* gene, with a clear family history of cancer. These new data provide more insight to support the role of *BRCA1* in CRC development.

Despite pancreatic cancer ranking fifth among five major GI cancers, its mortality and incidence rates are almost equal, making it an extremely deadly malignancy. Heritability is estimated to account for 21.5% of pancreatic cancer cases, thus it is important to identify the genetic and molecular background behind this population at risk. By performing a GWAS analysis and a recessive genetic model, Lu et al. identified a set of SNPs showing an association with pancreatic cancer risk (specific recessive effect; OR, 0.75-1.42, p<10^-5^). This study also emphasized the importance of choosing the right bioinformatic tool to improve the definition of polygenic risk scores.

For the treatment of most cancers, in particular for GI malignancies such as CRC, a combination of surgery with adjuvant therapy is widely used. However, the prognostic and risk factors for these patients have not yet been clarified. Furthermore, owing to a lack of effective markers, it is also not possible to infer their prognosis by only having data on genetic alterations in primary tumor tissue. To solve these problems, Yu et al. investigated the expression and protein levels of matrix metalloproteinase (MMP) gene family members. They found that a subgroup of MMP genes could have the potential to work as a target for precision therapies in CRC patients. More precisely, modifying their expression levels could significantly improve both progression-free survival and relapse-free time. In the same line of prognostic research, but for pancreatic adenocarcinomas (PAAD), Xu et al. investigated invasive-related genes with the aim of identifying different molecular subtypes of PAAD. Based on a molecular signatures analysis, a three-gene model was constructed and validated as a marker for assessing the prognostic risk of such patients.

Latest epidemiological data support the notion that at least 90% of cancer deaths from solid tumors are caused by metastases (5). Cancer metastases to the liver over a course of CRC occur in more than 50% of patients (6). To date, only a small number of cases have been candidates for a multimodal treatment, for which high drug resistance and relapse rates have been observed (7). As the mechanisms linked to those events are poorly understood, Kyrochristos et al. conducted a pilot study to assess the potential translational implications of intrapatient heterogeneity as a tool for defining appropriate matched drug therapy. Although it was a proof-of-concept approach, they provided strong evidence to support further evaluation, even in future clinical trials.

To fully understand GI cancer carcinogenesis, uncovering molecular data at each stage is essential. Several authors approached this topic by submitting review and original articles. Lv et al. described that, in CRC, Fibrillin-1 gene methylation can serve as a biomarker for disease development, as its transcriptional inactivation might start from normal colonic epithelium and increase through adenoma to CRC. Using a meta-analysis, Zhang et al. revealed that the Leptin G19A polymorphism may decrease the risk for CRC and esophageal GI cancers, as well as urinary tract cancer. Furthermore, Gao et al. demonstrated that a polymorphism (rs7158663) in the maternally expressed three-lncRNAs can be used as a genetic marker for predicting the risk of GI (CRC and gastric) and breast cancers. Finally, Mulder et al., in their submitted article, underlined that the development of orphan medicinal products for PAAD treatment should be closely monitored to increase the success rate of drugs reaching the market.

All of the collected articles support the notion that a deepened understanding of the molecular and genetic factors linked to the development of GI cancers could help to improve the translation of basic scientific research into clinical services and practices, pointing towards personalized medicine, and thus, more effective treatment of GI malignancies.

## Author contributions

All of the listed authors equally, substantially, and intellectually contributed to this Editorial and approved it for publication.
